# Harnessing psychoanalytical methods for a phenomenological neuroscience

**DOI:** 10.3389/fpsyg.2014.00334

**Published:** 2014-04-29

**Authors:** Emma P. Cusumano, Amir Raz

**Affiliations:** ^1^Department of Cognitive Science, McGill UniversityMontreal, QC, Canada; ^2^Department of Psychiatry, McGill UniversityMontreal, QC, Canada

**Keywords:** phenomenology of consciousness, phenomenology, first-person perspective, subjective experience, neuroscience methods

## Abstract

Psychoanalysis proffers a wealth of phenomenological tools to advance the study of consciousness. Techniques for elucidating the structures of subjective life are sorely lacking in the cognitive sciences; as such, experiential reporting techniques must rise to meet both complex theories of brain function and increasingly sophisticated neuroimaging technologies. Analysis may offer valuable methods for bridging the gap between first-person and third-person accounts of the mind. Using both systematic observational approaches alongside unstructured narrative interactions, psychoanalysts help patients articulate their experience and bring unconscious mental contents into awareness. Similar to seasoned meditators or phenomenologists, individuals who have undergone analysis are experts in discerning and describing their subjective experience, thus making them ideal candidates for neurophenomenology. Moreover, analytic techniques may provide a means of guiding untrained experimental participants to greater awareness of their mental continuum, as well as gathering subjective reports about fundamental yet elusive aspects of experience including selfhood, temporality, and inter-subjectivity. Mining psychoanalysis for its methodological innovations provides a fresh turn for the neuropsychoanalysis movement and cognitive science as a whole – showcasing the integrity of analysis alongside the irreducibility of human experience.

## INTRODUCTION

This paper illustrates how the marriage of phenomenology and psychoanalysis can inform the scientific study of consciousness. In particular, we outline the potential psychoanalysis holds as a tool for fostering different states of awareness and gathering experiential accounts for the purposes of cognitive neuroscience. Methods for elucidating the structures of phenomenal experience are scantily present in the landscape composing the cognitive sciences. This lacuna – a palpable gap between subjective and objective techniques – calls for expert methods to discern and describe experience from first and second person perspectives. While readily embracing psychodynamic theory, proponents of the neuropsychoanalysis movement have largely overlooked the methods inherent to analysis. A central aspect of the psychoanalytic approach, the unstructured narrative interaction forms the backbone of analysis. Though unconventional in the context of experimental neuropsychology, to disparage the narrative dynamic would cripple the research potential of psychoanalysis (Bazan, 2011). For example, cognitive scientists stand to benefit from narrative approaches to guide participants to uncover unconscious aspects of their experience, cultivate meta-awareness, and elicit descriptive firsthand reports. Here we argue that viewing psychoanalysis as a method for elucidating subjective experience best motivates collaboration between neuroscience and psychoanalysis. Sketching the crux of contemporary neuropsychoanalysis, we highlight the relative merits of a crosstalk with the critical neuroscience movement of neurophenomenology. We conclude by discussing how the development of new phenomenological techniques may leverage psychoanalytic methods in the clinical and experimental study of consciousness.

## NEUROPSYCHOANALYSIS IN FLUX

Neuroscientists, as well as psychoanalysts, are still trying to determine the nature of their collaboration in the burgeoning field of neuropsychoanalysis. Controversy regarding the relationship between psychoanalysis and the natural sciences dates back to Freud’s time, and continues to garner much attention today (Cohler and Galatzer-Levy, 2007). While some scholars consider neurobiology and psychoanalysis to be epistemologically and terminologically irreconcilable (Borch-Jacobsen and Shamdasani, 2011), the organic basis of mental life is one of the founding tenets of psychoanalysis (e.g., Freud, 1910b, p. 209). Freud (1895) had anticipated a future in which the psychological and neural sciences would coalesce. Since the 1990s, this vision has gradually come to fruition: while neuroscientists and cognitive psychologists have rekindled their interest in psychoanalytic ideas, analysts have increasingly turned toward the biological sciences (Fotopoulou et al., 2012). Overarching arguments continue to suggest that neuropsychoanalysis binds neuroscience and analysis by facilitating a crosstalk on topics of mutual interest (Solms and Turnbull, 2011). These global accounts inspire leading contemporary scholars to follow this intuitive lead and expound on the details of this cooperation.

Most research under the label of “neuropsychoanalysis” seeks to situate the concepts of psychoanalysis within the framework of contemporary neuroscience. Early collaboration, in the spirit of Freud, centered on understanding neuropathology from a psychodynamic perspective (Kaplan-Solms and Solms, 2000). This manner of clinically oriented investigation has since expanded to include studies of pathological behavior (e.g., depression and anxiety, Zellner et al., 2011), the neural mechanisms of psychodynamic psychotherapy (Gerber, 2011), as well as attempts to find biological measures for therapeutic outcomes (Shedler, 2010). Other research in neuropsychoanalysis fits with the preclinical cognitive neuroscience of consciousness. These investigations aim to develop models of the brain that accommodate and illuminate psychoanalytic phenomena such as repression (Bazan and Snodgrass, 2012), libido (Pfaff and Fisher, 2012), the dynamic unconscious (Shevrin et al., 1996; Berlin, 2011; Solms and Zellner, 2012), and dreaming (Zellner, 2013). As the investigatory domain of contemporary neuropsychoanalysis grows, so does the variety of empirical approaches: from neuroimaging techniques to experimental behavioral methods and animal studies (Fotopoulou et al., 2012).

Some analysts view neuropsychoanalysis as a weight on psychoanalytic discourse. Such clinicians see the movement as perpetuating the view that psychoanalysis needs biological bolstering to be complete, legitimate, and relevant (Blass and Carmeli, 2007). Other scholars have been especially critical of neuropsychoanalysis, suggesting that it could taint quality, and understanding of analysis among clinicians (Hoffman, 2009). These claims likely emerge in response to studies that purport to investigate the “scientific validity” of psychoanalytic theories (cf dream theory in Shirley, 2011). Alas, such scientism runs counter to the very epistemology set forth by the founders of neuropsychoanalysis, who encourage a balance between scientific objectivity and the subjective insights of psychoanalysis (Fotopoulou et al., 2012). Other critics argue that neuroscience is irrelevant to clinical practice, as the latter emphasizes uniquely personal accounts that are scantily amenable to scientific generalization (Pulver, 2003; Mechelli, 2010). Proponents of neuropsychoanalysis typically respond that while the entire spectrum of neuroscientific studies may be less clinically relevant to psychoanalysts, some studies undoubtedly are, for example animal studies that shed light on primal emotional behavior (Panksepp and Solms, 2012). And while concerns about the integrity of clinical practice remain an important issue, analysts also warn against an insular psychoanalysis that fails to engage in a constructive and critical dialog with the larger scientific community (Safran, 2012).

The collaboration between neuroscience and psychoanalysis reflects greater questions about the nature of scientific research, discourse, and validation (Aron, 2012). By incorporating phenomenological domains into traditional experimental paradigms, neuropsychoanalysis are reshaping the boundaries of science. Findings from neuroscience, moreover, increasingly govern our popular conceptions concerning behavior, psychopathology, and what it means to be human (Thornton, 2011; Young, 2011). Neuropsychoanalysis, therefore, joins the critical neuroscience movement in engaging the scientific community with discussions about culture, meaning, and the irreducibility of human experience (Choudhury and Slaby, 2011). Within this overarching search to unravel the relationship between the mind and body, a central question looms: can the objective third-person methods of cognitive science account for the first-person experience of subjective mental life?

## NEUROPHENOMENOLOGY AND THE PROBLEM OF CONSCIOUSNESS

The question of how and why humans are conscious – known amongst philosophers as the “hard problem” of consciousness – has provoked major debate concerning the nature of scientific inquiry. Most scientists posit that biological mechanisms subserve conscious experience, albeit little knowledge informs the details of such mechanisms (Chalmers, 1995, 2002). Neurologists have long recognized that we seem able to account for the workings of the human brain without recourse to influences beyond the laws of physical science (Eccles, 1965). Subsequently, many thinkers feel hard-pressed to speculate on the evolutionary and functional role of consciousness (Harnad, 2002). Such questions have led some philosophers to the conclusion that consciousness is an illusory heuristic (Churchland, 1981). These thinkers argue that conscious experience *just is* the neurobiological correlate of consciousness – a position that most neuroscientists hold as their “spontaneous philosophy” (Varela, 1998, p. 31; Dennett, 2001). Others disagree and argue that a description of mind that refers only to biological substrates and processes necessarily leaves something out. These scholars insist that consciousness is irreducible to information processing in the brain (Velmans, 2009) and emphasize that our conscious experience presupposes “every statement, model, or theory” of natural science (Thompson, 2004, p. 394). Such philosophical discord persists, reflecting and motivating practical challenges in the study of consciousness at the experimental level.

One response to the puzzle of consciousness comes from *neurophenomenology*, a movement founded by Varela et al. (1992). According to Varela, the hard problem is established on a deeply ingrained, falsely dualistic understanding of mind and brain. Instead of trying to philosophically “solve” the hard problem, he proposed that cognitive scientists take a methodological approach (Varela, 1996). In order to elucidate how and why experiences arise from neural processes, scientists require careful descriptions of experience to match the refined objective descriptions of brain functioning (Jack and Shallice, 2001; Lutz, 2002); however, researchers have largely eschewed such first-person methods. This trend partly owes to the recent focus in cognitive science on imaging of the living human brain alongside a long-standing ambivalence towards introspective reports (Nisbett and Wilson, 1977). Thus, while researchers are now equipped with many advanced methods for imaging and modeling the brain in fine-grained detail, sophisticated methods for describing and discriminating subjective experience continue to lag behind. Varela advocated bridging this methodological gap by incorporating the experiential techniques of phenomenology into a circulating dialog with the third-person methods of cognitive science. His neurophenomenology seeks to give an “explicit and central role to first-person accounts and to the irreducible nature of experience, while at the same time refusing both a dualistic concession or a pessimistic surrender” (Varela, 1998, p. 32). In doing so, neurophenomenology does not solve, but rather “dis-solves” the hard problem (Varela, 1998).

Proponents of Varela have realized the neurophenomenological project in different forms and under a variety of labels. Some scholars focus on the philosophical details of the collaboration between phenomenology and cognitive science (Gallagher, 2003). These theorists tease apart difficult phenomenological issues, such as the nature of indexing the self (Zahavi and Roepstorff, 2011), and conducting investigations into experiential realms pertinent to clinicians, including the phenomenology of psychopathology, (e.g., Sass et al., 2011). Neurophenomenology is also a growing scientific research program that seeks to modify and complement traditional methods of neuroscience for better phenomenological inquiry. For example, cognitive scientists have adapted a neuroimaging paradigm to allow for periodic “experience sampling” of subjects during experiments (Christoff et al., 2009). Some investigators utilize experimental participants who are already expert at observing and describing their experience, such as Buddhist meditators (Farb et al., 2007; Lutz et al., 2008). Other scholars have proposed that researchers may harness hypnotic and posthypnotic suggestion to alter subjective experience and encourage particular states of awareness (Lifshitz et al., 2013, 2014). One group of researchers in neurophenomenology focuses on developing first-person methods (Depraz et al., 2003; Vermersch, 2009) as well as second-person interviewing techniques for aiding subjects in attending to and articulating their experience (e.g., Petitmengin, 2006). Across all approaches, both theoretical and applied neurophenomenologists aim to bind first-person approaches with the techniques of modern cognitive science to uncover the basic structures of consciousness (Gallagher and Schmicking, 2010).

Psychoanalysis seems apposite to the goals of neurophenomenology; yet neurophenomenologists have largely overlooked psychoanalytic techniques and theories. Instead, neurophenomenologists have turned to two main sources of inspiration: Western Phenomenology (e.g., following philosophers such as Heidegger, Husserl, and Merleau-Ponty; see Schmicking, 2010) and Eastern contemplative traditions including Buddhist practices (Thompson, 2006; Schmicking, 2010). Research in neurophenomenology centers on training or guiding participants to discern and describe their experience with awareness and impartiality (Petitmengin and Bitbol, 2011). The neurophenomenology approach encourages individuals to reflexively observe consciousness from their first-person perspective and to recount accurate and detailed subjective reports (Lutz and Thompson, 2003). Although the inclusion of psychoanalysis in the neurophenomenological toolbox has yet to occur, Western Phenomenology, and psychoanalytic theory do overlap (Karlsson, 2010; Csordas, 2012). Indeed, the existential psychiatry movement of the early 20th century bridged the two disciplines in letter as well as in spirit (Halling and Dearborn Nill, 1995). Moreover, current efforts in neurophenomenology such as the “explicitation interview” harken to psychoanalytic techniques. The explicitation interview is a practice of “guided retrospective introspection” that incorporates inter-subjective guidance and non-leading suggestions to promote awareness of processes that typically remain implicit and un-seen within the field of experience, also known as “meta-awareness” (Maurel, 2009; Vermersch, 2009). Similarly, analysts describe the therapeutic process as “making the unconscious conscious” and commonly gage the completion of analysis by when the patient can freely articulate whatever comes to mind (Freud, 1910a; Wachtel, 2012). Thus, psychodynamic methods already suffuse established neurophenomenological protocols for cultivating meta-awareness in untrained participants.

The motives and objectives of neurophenomenology resonate with the call to neuropsychoanalysis. Both movements point to the need for a psychological theory in neuroscience that avoids fracturing or reducing human experience into a collection of functions and abilities. Just as neurophenomenologists emphasize the “embodied mind” (Clark, 1999; Thompson and Varela, 2001), neuropsychoanalysts urge scholars to reimagine the “minded brain” (Panksepp and Solms, 2012). Both groups argue that without a theoretical framework that operates at the level of the subject, neuropsychology fails to capture the psychological at all (Bazan, 2011), as well as stress the need for a “neurophenomenal level of analysis” in experimental neuropsychology (Panksepp and Solms, 2012). Like its phenomenological counterpart, neuropsychoanalysis can be seen as a direct effort against the implicit biological reductionism in cognitive neuroscience. Current research in the neuropsychoanalysis community appears biased toward the traditional methods of cognitive neuroscience. To analysts, collaboration with cognitive neuroscience runs the risk of reducing psychoanalytic “meaning” to neural “cause.” In line with the outlook Varela espoused, a turn toward the methodological in neuropsychoanalysis could help assuage these concerns, shifting the emphasis from psychoanalytic theory (e.g., testing the scientific validity of psychoanalytic concepts) to psychoanalytic methods (e.g., incorporating technical aspects of the therapeutic process into experimental protocols). However, while both neurophenomenology and neuropsychoanalysis call for a theory of experience at the subject level, only the former has generated an empirical program for incorporating that theory in practice. In closing, therefore, we propose that neuropsychoanalysis may present a new incarnation of neurophenomenology.

## CONCLUDING REMARKS

The practical realization of neuropsychoanalysis-as-neuropheno-menology presents many challenges. For example, is it possible to harness elements of the intuitive therapeutic process in an experimental context? Whereas Varela called upon Western phenomenology and Eastern contemplative traditions for their systematic treatment of firsthand experience, analysts often cite one of the hallmarks of psychoanalysis as the “imprecise” treatment of firsthand experience (Bazan, 2011, p. 2). Written accounts of therapeutic methods (e.g., the specific strategies that an analyst employs to bring out the unconscious stories of their patients) tend to take the form of case studies. Descriptions of more universal or underlying therapeutic techniques are sparse; analysts train *in vivo*, by engaging in clinical internships and undergoing analysis themselves. While philosophers and analysts have considered the mechanism of psychoanalytic insight and the relation between psychoanalytic insight and the structures of experience as described by cognitive and phenomenological science, these topics remain largely pristine on the proverbial “To Do” list of neuropsychoanalysis research (Strachey, 1934; Karlsson, 2010). And yet, phenomenology and psychoanalysis both draw on the premise that the vague nature of experience hardly precludes its careful articulation, analysis, and interpretation. A dialog between neuropsychoanalysis and neurophenomenology, therefore, would engender more precise ideas concerning the specific psychoanalytic techniques that can inform a correlation between firsthand descriptions of experience and third-person data.

One approach would advocate for including individuals who have undergone analysis as neurophenomenology participants. In the same way we treat seasoned meditators or phenomenologists, we can exploit the process of discerning and describing the psychoanalytic experience. This idea is scarcely new among analysts: “(One) might manipulate different neuropeptides, in research participants who are themselves psychoanalysts, and then have them describe their subjective states, using their expertise in doing so (with reference to the theoretical concepts that we use). Approaches such as this are rather radical, but they have huge potential, and appear to be remarkably underappreciated” (Solms and Turnbull, 2011, p. 9). To consider analysts and analysands experiential experts on par with, say, trained meditators raises many a problem. And yet, experiments involving either psychoanalytically trained individuals or Buddhist monks would both necessarily involve a second-person component – such as the explicitation interview – thereby exploiting a similar experimental approach.

Spanning an array of literature from clinical science and consciousness research, here we show how neurophenomenology casts a fresh light on the neuropsychoanalysis movement. While proponents of neuropsychoanalysis emphasize the importance of bringing a subject-oriented approach to cognitive neuroscience, these scholars have largely neglected the task of incorporating psychodynamic methods in an experimental setting. Cognitive scientists, however, stand to benefit from drawing on psychoanalytic techniques. Given their expertise in calling the unconscious mind to awareness, analysts could help researchers promote meta-awareness and gather subjective reports that effectively describe the structures of experience. At the same time, focusing on the possibilities of methodological exchange between neuroscience and psychoanalysis offers an answer to concerns within the neuropsychoanalysis community. Rather than foisting neuroscientific methods and models onto the theories of psychoanalysis, mining analysis for its phenomenological capabilities would ensure the integrity of the psychodynamic identity in a domain increasingly tinged with neuro-reductionism. Such an approach would allow neuropsychoanalysis to flourish because of, rather than despite, the different perspectives of its comprising disciplines.

## Conflict of Interest Statement

The authors declare that the research was conducted in the absence of any commercial or financial relationships that could be construed as a potential conflict of interest.

## References

[B1] Aron L. (2012). Rethinking “Doublethinking” psychoanalysis and scientific research – an introduction to a series. *Psychoanal. Dialogues* 22 704–709 10.1080/10481885.2012.733650

[B2] Bazan A. (2011). The grand challenge for psychoanalysis – and neuropsychoanalysis: taking on the game. *Front. Psychol.* 2:220 10.3389/fpsyg.2011.00220 PMC3171110 21941518

[B3] Bazan A. Snodgrass M. (2012). “On unconscious inhibition: instantiating repression in the brain,”in *From the Couch to the Lab: Trends in Psychodynamic Neuroscience* eds Fotopoulou A. Pfaff D. Conway M. A. (New York, NY: Oxford University Press), 307

[B4] Berlin H. A. (2011). The neural basis of the dynamic unconscious. *Neuropsychoanalysis* 13 5–31 10.1080/15294145.2011.10773654

[B5] Blass R. B Carmeli Z. V. I. (2007). The case against neuropsychoanalysis: on fallacies underlying psychoanalysis’ latest scientific trend and its negative impact on psychoanalytic discourse. *Int. J. Psychoanal.* 88 19–40 10.1516/6NCA-A4MA-MFQ7-0JTJ 17244565

[B6] Borch-Jacobsen M. Shamdasani S. (2011). *The Freud Files: An Inquiry Into the History of Psychoanalysis*. Cambridge, MA: Cambridge University Press

[B7] Chalmers D. J. (1995). Facing up to the problem of consciousness. *J. Conscious. Stud.* 2 200–219

[B8] Chalmers D. J. (2002). Consciousness and its place in nature. *Studies* 44 197–200

[B9] Choudhury S. Slaby J. (2011). *Critical Neuroscience: A Handbook of the Social and Cultural Contexts of Neuroscience*. Malden, MA: Wiley-Blackwell.

[B10] Christoff K. Gordon A. M. Smallwood J. Smith R. Schooler J. W. (2009). Experience sampling during fMRI reveals default network and executive system contributions to mind wandering. *Proc. Natl. Acad. Sci. U.S.A.* 106 8719–8724 10.1073/pnas.0900234106 19433790 PMC2689035

[B11] Churchland P. M. (1981). Eliminative materialism and the propositional attitudes. *J. Philos.* 78 67–90 10.2307/2025900

[B12] Clark A. (1999). An embodied cognitive science? *Trends Cogn. Sci.* 3 345–351 10.1016/S1364-6613(99)01361-3 10461197

[B13] Cohler B. J. Galatzer-Levy R. (2007). What kind of science is psychoanalysis? *Psychoanal. Inq.* 27 547–582 10.1080/07351690701468108

[B14] Csordas T. J. (2012). Psychoanalysis and phenomenology. *Ethos* 40 54–74 10.1111/j.1548-1352.2011.01231.x

[B15] Dennett D. C. (2001). The fantasy of first-person science. *Debate Conducted with David Chalmers at the Northwestern University, Evanston, IL.* Available at: http://ase.tufts.edu/cogstud/dennett/papers/chalmersdeb3dft.htm (accessed February 15, 2001)

[B16] Depraz N. Varela F. J. Vermersch P. (2003). *On Becoming Aware: A Pragmatics of Experiencing*. Philadelphia, PA: John Benjamins Publishing Company. 10.1075/aicr.43

[B17] Eccles J.C. (1965). “Discussion after ‘Consciousness’,” in *Brain and Conscious Experience: Study Week of The Pontificia Academia Scientiarum* ed. Eccles J. C. (New York, NY: Springer-Verlag)

[B18] Farb N. A. Segal Z. V. Mayberg H. Bean J. Mckeon D. Fatima Z. (2007). Attending to the present: mindfulness meditation reveals distinct neural modes of self-reference. *Soc. Cogn. Affect. Neurosci.* 2 313–322 10.1093/scan/nsm030 18985137 PMC2566754

[B19] Fotopoulou A. Pfaff D. Conway M. A. (2012). *From the Couch to the Lab: Trends in Psychodynamic Neuroscience*. New York, NY: Oxford University Press 10.1093/med/9780199600526.001.0001

[B20] Freud S. (1895). “Project for a scientific psychology (1950 [1895]),” in *The Standard Edition of the Complete Psychological Works of Sigmund Freud Vol. 1 (1886–1899): Pre-Psycho-Analytic Publications and Unpublished Drafts* eds Strachey J. Freud A. (London: Hogarth) 281–391

[B21] Freud S. (1910a). Five lectures on psychoanalysis. *Am. J. Psychol.* 21 181–218 10.2307/1413001

[B22] Freud S. (1910b). “On narcissism: an introduction,” in *The Standard Edition of the Complete Psychological Works of Sigmund Freud, Vol. 14, (1914–1916): On the History of the Psycho-Analytic Movement, Papers on Metapsychology and Other Works* eds Strachey J. Freud A. (London: Hogarth) 67–102

[B23] Gallagher S. (2003). Phenomenology and experimental design toward a phenomenologically enlightened experimental science. *J. Conscious. Stud.* 10 9–10

[B24] Gallagher S. Schmicking D. (2010). *Handbook of Phenomenology and Cognitive Science*. New York, NY: Springer

[B25] Gerber A.J. (2011). “Commentary: neurobiology of psychotherapy – state of the art and future directions,” in *Psychodynamic Psychotherapy Research: Evidence-Based Practice and Practice-Based Evidence* eds Levy R. A. Ablon J. S. Chele H. K. (New York: Springer)

[B26] Halling S Dearborn Nill J. (1995). A brief history of existential-phenomenological psychiatry and psychotherapy. *J. Phenomenol. Psychol.* 26 1–45 10.1163/156916295X00024

[B27] Harnad S. (2002). “Turing indistinguishability and the blind watchmaker,” in *Consciousness Evolving* ed. Fetzer J. (Philadelphia, PA: John Benjamins) 3–20

[B28] Hoffman I. Z. (2009). Doublethinking our way to ‘scientific’ legitimacy: the desiccation of human experience. *J. Am. Psychoanal. Assoc.* 57 1043–1069 10.1075/aicr.34.04har 19837853

[B29] Jack A. I. Shallice T. (2001). Introspective physicalism as an approach to the science of consciousness. *Cognition* 79 161–196 10.1177/0003065109343925 11164027

[B30] Kaplan-Solms K. Solms M. (2000). *Clinical Studies in Neuro Psychoanalysis*. London: Karnac Books. 10.1016/S0010-0277(00)00128-1

[B31] Karlsson G. (2010). *Psychoanalysis in a New Light*. Cambridge: Cambridge University Press. 10.1017/CBO9780511845147

[B32] Lifshitz M. Cusumano E. P. Raz A. (2013). Hypnosis as neurophenomenology. *Front. Hum. Neurosci.* 7:469 10.3389/fnhum.2013.00469 PMC3744032 23966930

[B33] Lifshitz M. Cusumano E. P. Raz A. (2014). “Meditation and hypnosis at the intersection between phenomenology and cognitive science,” in *Meditation–Neuroscientific Approaches and Philosophical Implications* eds Schmidt S. Walach H. (New York, NY:Springer) 211–226 10.1007/978-3-319-01634-4_12

[B34] Lutz A. (2002). Toward a neurophenomenology as an account of generative passages: a first empirical case study. *Phenomenol. Cogn. Sci.* 1 133–167 10.1023/A:1020320221083

[B35] Lutz A. Slagter H. A. Dunne J. D. Davidson R. J. (2008). Attention regulation and monitoring in meditation. *Trends Cogn. Sci.* 12 163–169 10.1016/j.tics.2008.01.005 18329323 PMC2693206

[B36] Lutz A. Thompson E. (2003). Neurophenomenology integrating subjective experience and brain dynamics in the neuroscience of consciousness. *J. Conscious. Stud.* 10 9–10

[B37] Maurel M. (2009). “The explicitation interview: examples and applications,” in *Ten Years of Viewing from Within: The Legacy of Francisco Varela* ed. Petitmengin C. (Charlottesville, VA: Imprint Academic) 58–89

[B38] Mechelli A. (2010). Psychoanalysis on the couch: can neuroscience provide the answers? *Med. Hypotheses* 75 594–599 10.1016/j.mehy.2010.07.042 20709458

[B39] Nisbett R. E. Wilson T. D. (1977). Telling more than we can know: verbal reports on mental processes. *Psychol. Rev.* 84 231 10.1037/0033-295X.84.3.231

[B40] Panksepp J. Solms M. (2012). What is neuropsychoanalysis? Clinically relevant studies of the minded brain. *Trends Cogn. Sci.* 16 6–8 10.1016/j.tics.2011.11.005 22153583

[B41] Petitmengin C. (2006). Describing one’s subjective experience in the second person: an interview method for the science of consciousness. *Phenomenol. Cogn. Sci.* 5 229–269 10.1007/s11097-006-9022-2

[B42] Petitmengin C. Bitbol M. (2011). Lets trust the (Skilled) subject! A reply to Froese, Gould and Seth. *J. Conscious. Stud.* 18 90–97

[B43] Pfaff D. W. Fisher H. E. (2012). “Generalized brain arousal mechanisms and other biological, environmental, and psychological mechanisms that contribute to libido,”in *From the Couch to the Lab: Trends in Psychodynamic Neuroscience* eds Fotopoulou A. Pfaff D. Conway M. A. (New York, NY: Oxford University Press) 64

[B44] Pulver S. E. (2003). On the astonishing clinical irrelevance of neuroscience. *J. Am. Psychoanal. Assoc.* 51 755–772 10.1177/00030651030510032101 14596560

[B45] Safran J. D. (2012). Doublethinking or dialectical thinking: a critical appreciation of Hoffman’s ‘Doublethinking’ critique. *Psychoanal. Dialogues* 22 710–720 10.1080/10481885.2012.733655

[B46] Sass L. Parnas J. Zahavi D. (2011). Phenomenological psychopathology and schizophrenia: contemporary approaches and misunderstandings. *Philos. Psychiatr. Psychol.* 18 1–23 10.1353/ppp.2011.0008

[B47] Schmicking D. (2010). “A toolbox of phenomenological methods,” in *Handbook of Phenomenology and Cognitive Science* eds Gallagher S. Schmicking D. (New York, NY: Springer) 35–55

[B48] Shedler J. (2010). The efficacy of psychodynamic psychotherapy. *Am. Psychol.* 65 98–109 20141265 10.1037/a0018378

[B49] Shevrin H. Bond J. A. Brakel L. A. W. Hertel R. K. Williams W. J. (1996). *Conscious and Unconscious Processes: Psychodynamic, Cognitive, and Neurophysiological Convergences*. New York, NY: Guilford Press. 10.1037/a0018378

[B50] Shirley A. (2011). The scientific status of psychoanalytic dream theory. *Br. J. Med. Psychol.* 43 13–17 10.1111/j.2044-8341.1970.tb02097.x 5530531

[B51] Solms M. Turnbull O. H. (2011). What is neuropsychoanalysis? *Neuropsychoanalysis* 13 133–146 10.1080/15294145.2011.10773670

[B52] Solms M. Zellner M. R. (2012). “The freudian unconscious today,” in *From the Couch to the Lab: Trends in Psychodynamic Neuroscience* eds Fotopoulou A. Pfaff D. Conway M. A. (New York, NY: Oxford University Press) 209

[B53] Strachey J. (1934). The nature of the therapeutic action of psychoanalysis. *Int. J. Psychoanal.* 15 127–159

[B54] Thompson E. (2004). Life and mind: from autopoiesis to neurophenomenology. A tribute to Francisco Varela. *Phenomenol. Cogn. Sci.* 3 381–398 10.1023/B:PHEN.0000048936.73339.dd

[B55] Thompson E. (2006). “Neurophenomenology and contemplative experience,” in *The Oxford Handbook of Religion and Science* eds Clayton P. Simpson Z. (New York, NY: Oxford) 226–235 10.1093/oxfordhb/9780199279272.003.0015

[B56] Thompson E. Varela F. J. (2001). Radical embodiment: neural dynamics and consciousness. *Trends Cogn. Sci.* 5 418–425 10.1016/S1364-6613(00)01750-2 11707380

[B57] Thornton D. J. (2011). *Brain Culture: Neuroscience and Popular Media*. New Brunswick, NJ: Rutgers University Press

[B58] Varela F. J. (1996). Neurophenomenology: a methodological remedy for the hard problem. *J. Conscious. Stud.* 3 330–349

[B59] Varela F. J. (1998). “A science of consciousness as if experience mattered,” in *Towards a Science of Consciousness II: The Second Tucson Discussion and Debates* eds Hameroff S. Kaszniak A. W. Scott A. C. (Cambridge, MA: MIT Press) 31–44

[B60] Varela F. J. Thompson E. T. Rosch E. (1992). *The Embodied Mind: Cognitive Science and Human Experience*. Cambridge, MA: MIT press

[B61] Velmans M. (2009). *Understanding Consciousness*: 2nd Edn New York, NY: Taylor & Francis Group

[B62] Vermersch P. (2009). Describing the practice of introspection. *J. Conscious. Stud.* 16 10–12

[B63] Wachtel P. L. (2012). Reflections on the therapeutic process. *Psychoanal. Perspect.* 9 88–117 10.1080/1551806X.2012.662101

[B64] Young A. (2011). Self, brain, microbe, and the vanishing commissar. *Sci. Technol. Hum. Values* 36 638–661 10.1177/0162243910388024

[B65] Zahavi D. Roepstorff A. (2011). Faces and ascriptions: mapping measures of the self. *Conscious. Cogn.* 20 141–148 10.1016/j.concog.2010.10.011 21075009

[B66] Zellner M. R. (2013). Dreaming and the default mode network: some psychoanalytic notes. *Contemp. Psychoanal.* 49 226–232 10.1080/00107530.2013.10746548

[B67] Zellner M. R. Watt D. F. Solms M. Panksepp J. (2011). Affective neuroscientific and neuropsychoanalytic approaches to two intractable psychiatric problems: why depression feels so bad and what addicts really want. *Neurosci. Biobehav. Rev.* 35 2000–2008 10.1016/j.neubiorev.2011.01.003 21241736

